# Using patient management as a surrogate for patient health outcomes in diagnostic test evaluation

**DOI:** 10.1186/1471-2288-12-12

**Published:** 2012-02-14

**Authors:** Lukas P Staub, Sarah J Lord, R John Simes, Suzanne Dyer, Nehmat Houssami, Robert YM Chen, Les Irwig

**Affiliations:** 1 NHMRC Clinical Trials Centre, The University of Sydney, Sydney, Australia; 2 The Screening and Test Evaluation Program, The University of Sydney, Sydney, Australia; 3 St. Vincent's Hospital, Melbourne, Australia

## Abstract

**Background:**

Before a new test is introduced in clinical practice, evidence is needed to demonstrate that its use will lead to improvements in patient health outcomes. Studies reporting test accuracy may not be sufficient, and clinical trials of tests that measure patient health outcomes are rarely feasible. Therefore, the consequences of testing on patient management are often investigated as an intermediate step in the pathway. There is a lack of guidance on the interpretation of this evidence, and patient management studies often neglect a discussion of the limitations of measuring patient management as a surrogate for health outcomes.

**Methods:**

We discuss the rationale for measuring patient management, describe the common study designs and provide guidance about how this evidence should be reported.

**Results:**

Interpretation of patient management studies relies on the condition that patient management is a valid surrogate for downstream patient benefits. This condition presupposes two critical assumptions: the test improves diagnostic accuracy; and the measured changes in patient management improve patient health outcomes. The validity of this evidence depends on the certainty around these critical assumptions and the ability of the study design to minimise bias. Three common designs are test RCTs that measure patient management as a primary endpoint, diagnostic before-after studies that compare planned patient management before and after testing, and accuracy studies that are extended to report on the actual treatment or further tests received following a positive and negative test result.

**Conclusions:**

Patient management can be measured as a surrogate outcome for test evaluation if its limitations are recognised. The potential consequences of a positive and negative test result on patient management should be pre-specified and the potential patient benefits of these management changes clearly stated. Randomised comparisons will provide higher quality evidence about differences in patient management using the new test than observational studies. Regardless of the study design used, the critical assumption that patient management is a valid surrogate for downstream patient benefits or harms must be discussed in these studies.

## Background

Before a new test is introduced in clinical practice, evidence is needed to demonstrate that its use will lead to improvements in patient health outcomes [1]. Studies reporting test accuracy may not be sufficient, and clinical trials of tests that follow patients over the whole pathway from testing to treatment outcomes, although ideal, are rarely feasible [2]. Therefore, studies investigating the consequences of tests on patient management, that is the use of treatment and the ordering of further tests, are sometimes undertaken to support conclusions about test effectiveness. The rationale for these studies is that a test must change patient management to provide health benefits, or at least not compromise management while providing other benefits, such as improved safety. Interpretation of these studies, however, presents the fundamental problem that a measured change in management does not necessarily translate to improved health outcomes.

The obstacles of using management as a surrogate for health outcomes were described by Fineberg and colleagues [3] in their 1977 study of cranial Computed Tomography (CT), one of the first of its kind. They explained that the impact of CT on therapeutic plans is only a "way-station measure of clinical efficacy on the path to patient outcome". Guyatt et al.'s landmark paper on the use of diagnostic before-after studies, which compare planned patient management before and after testing, provided structured guidance for researchers about the methodological challenges of patient management studies [4]. However, a search of the literature indicates that little methodological work has been done in this field over the last 25 years. Some guidance exists about the design [5] and appraisal [6] of diagnostic before-after studies, but there are no guidelines for researchers on different types of study design and the interpretation of change in management as a surrogate for health outcomes.

Unsurprisingly perhaps, there is little consistency in how evidence about the impact of testing on patient management is reported and interpreted in the literature. For example, a study about Positron Emission Tomography (PET) for various cancers, designed to support recommendations for funding, briefly mentions that the intended changes in management measured do not always go in the correct direction and improve outcomes [7]. Other current examples, such as studies of the impact of prognostic markers on the selection of adjuvant cancer treatments [8], do not caution about the limitations of this evidence.

Methodological guidance on this topic is an important area of need for researchers, clinicians and others making decisions about the use of tests. A systematic review of health technology assessments (HTAs) of medical tests published between 2005 and 2010 demonstrated that the impact of testing on patient management is frequently assessed, with evidence available from a range of different study designs including randomised trials, but interpretation of this evidence for conclusions about the impact of the test on health outcomes is inconsistently reported [9].

Although researchers can follow standard epidemiological principles for intervention studies when designing studies to measure the effect of a test on patient management, the interpretation of this endpoint as a surrogate for health outcomes raises different unique issues. In this paper, we explain how the validity of this evidence depends on both the level of certainty surrounding assumptions linking changes in patient management to improved health outcomes and the susceptibility of different study designs to biases.

## Methods

In the absence of guidelines, we developed the ideas presented in this paper with Fryback and Thornbury's widely adopted 6-level hierarchical framework as a starting point [10]. Several modifications of this framework have been published [11]. Most of these variations share the same basic hierarchy to rank test evidence according to the type of outcomes evaluated along the causal pathway linking testing with treatment outcomes. Together with test accuracy, they recognise impact on patient management as an intermediate outcome that is necessary but not sufficient alone to infer change in patient health outcomes.

For the purpose of this paper, we defined patient management studies as an empirical study designed to measure whether information provided by a medical test changes clinician decisions about treatment or further investigation.

Following the GRADE system for judging the quality of evidence for medical tests in terms of their consequences on patient outcomes [1], we identified two major issues that need to be explored when interpreting patient management studies: the validity of assumptions that changes in patient management lead to improved health outcomes, which are independent of study design, and the avoidance of bias in the estimates of patient management, which depends on study design. These issues are dealt with in turn in the following sections. We first explain the guiding principles for using patient management as a surrogate outcome, followed by a description of the different study designs and key points for interpretation of results.

## Results

### Patient management as a surrogate outcome

**Figure 1:**
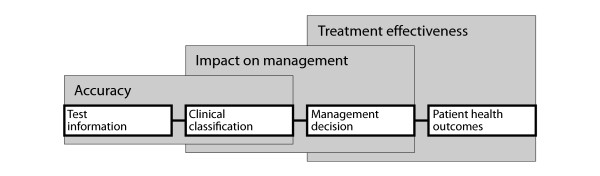
**Test-treatment pathway showing Accuracy, Impact on management and Treatment effectiveness as determinants of health outcomes**. Adapted from Staub et al. [9]

The Fryback and Thornbury framework [10] recognises that medical tests are embedded in a clinical pathway. We use a simplified version of the clinical pathway, referred to here as the 'test-treatment pathway', to illustrate the key concepts (Figure 1). This pathway is characterised by the population who will be tested, and the available tests and management options. It shows that when a test is claimed to improve patient health outcomes by improving management decisions, the key determinants are test accuracy for detecting cases eligible for a change in management and the effectiveness of the management. Measuring patient management as a surrogate outcome involves specifying what changes in management will occur that are expected to benefit patients, rather than measuring overall changes in management. The proposed role of a test within the pathway, that is whether it will be used as a replacement for an existing test, as an add-on after existing tests, or before as a triage test to achieve this claim also needs to be stated [12].

### Critical assumptions

By definition, patient management studies do not provide direct evidence about health outcomes. Interpretation relies on the condition that the outcome measured, for example surgery performed following a true (or false) positive test result, is a valid surrogate for downstream patient benefits (or harms).

This condition presupposes two critical assumptions: First, the test improves accuracy, which is important in order to judge whether changes in patient management are likely to be based on correct test results. Comparative diagnostic accuracy studies of the existing and new tests are needed to provide this evidence [12]. These studies investigate the veracity of test results by comparing the tests with a reference standard. They provide estimates of the true positive rate (sensitivity) and true negative rate (specificity) of a test [13].

The second assumption is that the changes in patient management observed improve patient health outcomes. Judging whether this assumption is reasonable may sometimes be straightforward, for example if the test reduces the use of unnecessary invasive tests, but is usually more complicated and relies on careful assessment of supportive evidence. This assessment requires expert opinion. Two common scenarios are discussed below.

### Two common scenarios

**Figure 2:**
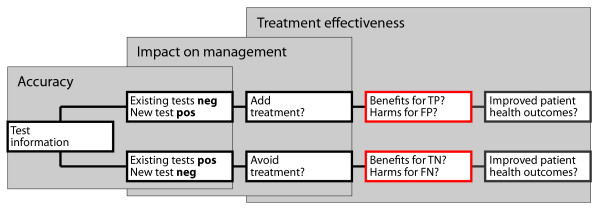
**Identifying critical assumptions that changes in patient management improve patient health outcomes**. Abbreviations: pos = positive, neg = negative, TP = true positive, FP = false positive, TN = true negative, FN = false negative

In the first scenario, the new test is proposed to improve outcomes by adding treatment for patients testing positive who would not have been detected by existing tests (Figure 2, top). Judgements are required that treatment is effective for the additional true positive findings, and these benefits outweigh the harms of unnecessary treatment or further testing in false positives.

If previous trials have demonstrated treatment is effective for patients identified using existing tests, it may be possible to extrapolate these findings to the additional cases detected by the new test [14]. Unfortunately, there is no simple algorithm to assess generalisability; therefore clinical judgement is required. Importantly, the additional cases are likely to represent a different disease spectrum to cases detected by the existing tests alone. Based on the principles used to consider whether trial evidence can be applied to an individual patient [15], two necessary assumptions are: i) the baseline risk of disease events in the new population is high enough to warrant treatment, and ii) existing trial evidence about treatment effectiveness can be applied to this population. The latter may be considered reasonable if trials have demonstrated that the relative treatment effects are similar across a broad disease spectrum. If so, the estimated treatment benefits then need to be weighed up against the potential harms of false positive findings (Table 1). However, if a new test detects cases earlier in the disease process, there may not be sufficient existing evidence that can be used to extrapolate patient benefits.

**Table 1:** Judging whether a change in patient management is likely to improve patient health outcomes

Test claim	Improved health outcomes				Conclusion	Example
	
	Assumption 1		Assumption 2			
	
	Test accuracy	Supportive evidence	Impact of changed management on health outcomes	Supportive evidence		
Adds effective treatment by detecting more cases (where new test pos, existing tests neg)	New test is more sensitive than existing tests (more TP cases)	Comparative accuracy study	**Benefits for TP** Absolute treatment benefits outweigh harms: i) risk of disease events is clinically important, and	i) study measuring prognosis in test-stratified population	**Judgement** Evidence supports potential benefits of treatment for TP, and these benefits outweigh harms for FP Or Require RCT	Breast MRI added to conventional imaging
			ii) relative treatment effects for TP detected by existing tests also apply to additional TP detected by new test	ii) RCT measuring treatment effectiveness for disease subgroups		
			**Harms for FP** iii) harms of unnecessary treatment are acceptable	iii) study measuring risk of treatment adverse events		

Avoids unnecessary treatment or further tests by excluding more non-cases (where new test neg, existing tests pos)	New test is more specific than existing tests (fewer FP cases)	Comparative accuracy study	**Benefits for TN** i) treatment or further tests carry a risk of adverse events	i) study measuring risk of treatment adverse events	**Judgement** Evidence supports benefits of avoiding treatment or further tests for TN outweigh potential harms for FN cases Or Require RCT	POC prothrombin time versus clinical judgement
			**Harms for FN** ii) harms of omitting/delaying treatment in FN cases are acceptable	ii) study measuring prognosis in test negative patients not receiving treatment		

Abbreviations: pos = positive, neg = negative, TP = true positive, FP = false positive, TN = true negative, FN = false negative, RCT = randomised controlled trial, MRI = magnetic resonance imaging, POC = Point-of-care

To illustrate, a systematic review has shown that Magnetic Resonance Imaging (MRI) is more sensitive in detecting additional breast cancer foci which are occult on conventional imaging [16]. The authors concluded that women with true positive MRI findings often receive more extensive surgery and may potentially benefit from better local tumour control, whereas those with false positive results have additional biopsies which may delay treatment and/or result in more breast tissue excised than necessary, including unnecessary mastectomy, leading to poorer cosmetic outcomes. We suggest it would be helpful to include an explicit statement saying that inferences about the benefits of more extensive surgery in this MRI classified population would be strengthened by evidence that this population has a higher risk of local recurrence than patients testing negative and that the relative benefits of more extensive surgery are similar for patients with varying extent of disease. When there is doubt (and clinical equipoise) about assumptions of improved health outcomes, randomised trials of treatments in the uncertain subgroups are recommended.

In the second scenario, the new test is proposed to improve patient outcomes by avoiding unnecessary treatment for patients testing negative who would have been classified as positive by existing tests (Figure 2, bottom). Judgements are required that the benefits of avoiding unnecessary treatment in true negative findings outweigh the harms of missed or delayed treatment in false negatives. The same concepts apply when the new test is proposed to avoid further testing rather than changing treatment. The benefits of avoiding management in true negative findings can be estimated by considering the risk of adverse events and costs associated with the treatment (or further testing) that would not result in any benefits for the patient.

Information about the harms of avoiding management following a false negative result is usually less readily available. Studies may be required to demonstrate that the risk of adverse events in patients testing negative is below an acceptable level to infer low risk of harm from false negatives (Table 1). This can be measured in single-arm studies of tested patients or trials that compare the new test strategy with existing tests. Such trials can also assess the trade-off between benefits and harms. For example, point-of-care prothrombin time testing has been shown to be highly accurate in providing test results to anaesthesiologists, with minimum delay in the operating theatre, to make decisions about the use of blood products in bleeding patients [17]. The investigators concluded that point-of-care prothrombin time testing has the potential "to improve patient morbidity and mortality by: reducing the rate of unnecessary transfusion of fresh frozen plasma and associated complications in patients testing negative, and reducing major intra-operative bleeding through earlier detection and more rapid treatment of coagulopathic patients". False negative test results are not expected to have a major detrimental effect, because a negative test would shift rather than replace the clinical threshold for transfusion, so patients who clearly demonstrate abnormal bleeding would still receive blood products regardless of a negative test result. However, it was also concluded that these assumptions of the impact of the test on patient management and subsequent outcomes needed confirmation, and a trial investigating these questions is underway [18].

In a third possible scenario, the number of patients receiving the specified management remains the same but the new test has other attributes, for example it is less invasive or less expensive than the existing tests, with similar accuracy. Conclusions of no change in management essentially require an assessment of equivalence to exclude a clinically important difference in patient management when using the new or existing test strategies. This type of study may also be useful to provide evidence that an old test is redundant and removing it does not compromise patient management.

### Estimating the magnitude of changes in patient management

A test result is only one of several factors influencing patient management. Therefore, management studies are designed to estimate the proportion of patients in whom the findings from the new test translate to an actual change in management. This quantification allows researchers to measure and weigh up the consequences of true and false test results for patients.

By estimating the magnitude of changed management, these studies demonstrate the 'activity' of the test on changing management. The review of breast MRI showed that a proportion, but not all, additional positive MRI findings lead to more extensive surgery [16]. Evidently some of these cases do not meet the decision threshold for an increase in treatment. The important issue, however, is that we are not sure whether more extensive surgery improves patient health outcomes [19].

A study that finds no management change may still reveal other important attributes of a test outside the test-treatment pathway, such as reassurance or reduced test failure rates. A trial that assessed the impact of MRI and CT in patients with low back pain showed that imaging significantly increased diagnostic confidence without changing treatment plans [20]. Even so, slightly improved outcomes have been reported in back pain patients undergoing imaging, which were attributed to the direct test effect of reassurance rather than indirect effects through management changes [21].

Patient management studies can also address other research questions, for example to audit clinician adherence to clinical guidelines. For example, a trial of the Canadian CT Head Rule showed that the use of this decision aid did not reduce the rates of CT imaging in Canadian emergency departments [22].

### Study designs

Both experimental and observational study designs can be used to measure change in patient management. The quality of the evidence from these studies depends on how well they are designed to minimise bias. Studies reporting changes in actual management provide higher quality evidence than studies relying on hypothetical planned management.

**Figure 3:**
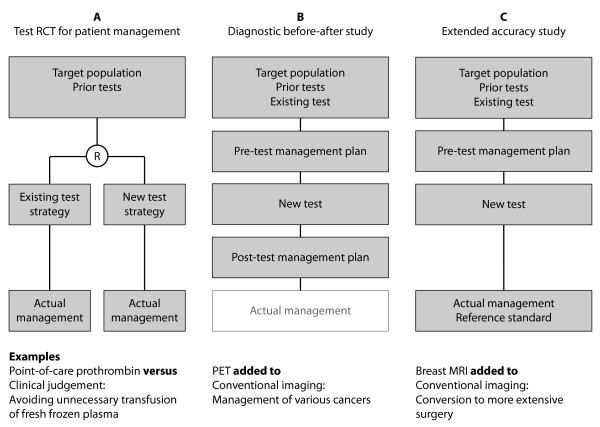
**Study designs to assess the impact of tests on patient management**. Abbreviations: RCT = randomised controlled trial, R = randomise, PET = positron emission tomography, MRI = magnetic resonance imaging

RCTs with patient management as a primary endpoint are the optimal design to minimise bias for measurement of the consequences of alternative test strategies (Figure 3A), because they do not rely on assumptions of planned management. Such RCTs are suitable for replacement, add-on or triage tests and can also measure any other important intermediate consequences of the test procedure, such as test safety or patient acceptability. This evidence may be instrumental in the planning of definitive trials of a test. Rules developed for the design and reporting of treatment trials also apply to trials reporting on patient management [23]. An example is the ongoing POC-OP trial, which measures the effectiveness of point-of-care prothrombin time testing to reduce the administration of blood products during and after surgery [18]. Patients are randomised to usual care plus point-of-care testing or usual care only, and the primary outcome is the relative risk of receiving any fresh frozen plasma perioperatively. If point-of-care prothrombin time testing in the operation theatre proves to reduce the administration of fresh frozen plasma, this may lead to decreased costs and avoid complications associated with the administration of allogenic blood products.

Despite the important advantages of using randomised comparisons, the most common type of study used to measure the impact of test results on patient management is the observational 'diagnostic before-after' study (Figure 3B) [4]. These studies are often used to provide an overview of the impact of test results on patient management in broad patient groups where multiple differential diagnoses are considered. Pre- and post-test management plans, both hypothetical, are recorded to estimate the potential impact of test results on patient management. These studies can only be applied to add-on tests and are prone to bias, foremost because clinicians may not use the same caution when defining pre-test management plans, for example the intent to perform surgery, as they do in real-life practice because they know these plans can be revised once the test results are available. Measuring actual management in addition to hypothetical post-test management plans can assess the extent to which planned management is put into practice. Fineberg and colleagues used this design to examine patient management in the study of cranial CT [3]. Similar studies are found in the current medical literature to support conclusions about the effectiveness of PET for various cancers [24-27]. In the United States, the National Oncologic PET Registry was established to show whether PET has a similar impact on intended patient management in previously unfunded rare cancer types, and to identify variations in the types of management changes between different malignancies [28]. PET changed the planned treatment or no-treatment decision in more than a third of tested patients. PET more commonly led to cancer upstaging due to the delineation of a greater tumour mass than downstaging. If the initial plan was a biopsy, potentially three quarters of these biopsies might be avoided due to PET [25]. The authors concluded that, in view of its consistent impact for a wide range of cancers, the use of PET should not be restricted by cancer type [26].

A related design is epidemiological studies that compare the management of patients in current practice, which includes the information of a new test, with the management of historic controls before the test was used. For example, the introduction of coronary CT has been shown to reduce the downstream use of further tests compared to a period before the test was available [29].

Finally, test accuracy studies are sometimes extended to report on the actual management following testing when it is uncertain whether true or false additional findings from the test lead to a change from the hypothetical pre-test management plan (Figure 3C). This is only possible when measurement of the reference standard and patient management do not influence each other, that is if the reference standard can be performed at or directly after management, or before management if it is ethical to blind clinicians to the results of the reference standard, for example in situations where the results will not be available within the time frame required for clinical decisions. Extended accuracy studies can only be used for add-on tests where the pre-test management plan is clearly determined from the results of the existing test. Guidelines developed for the design and reporting of classical accuracy studies also apply [13]. In the example of breast MRI, this test is used for the staging of localised breast cancer to detect additional foci that are occult on conventional imaging. It has been promoted to improve treatment by identifying women who may benefit from a conversion from wide local excision to more extensive surgery. As mentioned above, a systematic review has confirmed the higher sensitivity of breast MRI compared with conventional imaging [16]. Some accuracy studies included in this review also reported on patient management, providing evidence that some, but not all, additional true positive MRI findings lead to more extensive surgery. On the other hand, some patients received more extensive surgery unnecessarily due to false positive MRI results. The authors concluded that women with true positive MRI findings who receive more extensive surgery may potentially benefit from better local tumour control, but trials are needed to confirm these benefits against the use of standard adjuvant therapy; whereas those with false positive results have additional biopsies which may delay treatment and/or result in more breast tissue excised than necessary, which may lead to a poorer postoperative cosmetic outcome. A more recent RCT measuring re-excision rates as a primary endpoint has now reported that the addition of breast MRI does not reduce reoperation rates [30].

## Conclusions

Patient management can be measured as a surrogate outcome for test evaluation if its limitations are recognised. This evidence can support recommendations about the use of a test when assumptions about changes in management associated with the test are uncertain and pivotal to conclusions. It can also be incorporated in decision analysis models designed to weigh up the potential benefits of a test against its potential harms and to inform cost-effectiveness analyses.

To date, there are no accepted guidelines for the design, reporting and appraisal of patient management studies. Appraisal tools for test accuracy studies have been adapted to assess the quality of diagnostic before-after studies [6]; however, readers need to be able to decide whether patient management is a valid surrogate for patient health outcomes. Below, we list some principles to guide clinical researchers toward more transparent reporting and valid interpretation of these studies.

• Studies of patient management must provide a clear description of the role and position of a test in the clinical pathway, to judge whether the study results are applicable to the way they are intended to be used in practice.

• The potential consequences of a positive and negative test result on patient management need to be pre-specified and the potential patient benefits of these management changes clearly stated.

• Randomised comparisons will provide higher quality evidence about differences in patient management using the new test than observational studies.

• A detailed description of the study design is crucial to understand how the authors attempted to minimise bias, and to decide whether the study results are robust.

• Researchers need to report information on how the data were collected for a study. If actual management was not recorded, were management plans prospectively reported on dedicated case report forms or only assumed based on information from patient charts?

• The estimates of changes in management must be reported contingent on test results; otherwise it is not possible to conclude to what extent management changes are dependent on the test.

• Patient management studies are easiest to interpret if it can be assumed that clinicians use all test information appropriately in a standard way, so that a change in patient management can be attributed to the test itself rather than to variations in the clinicians' behaviour. If variations between clinicians are anticipated, for example due to differences in preference or experience, these factors can also be measured to assist interpretation of the results.

In conclusion, researchers should provide clear statements about the assumptions made regarding the effect of changes in management on patient health outcomes. These assumptions should be based on a discussion of the availability and quality of existing evidence for both test accuracy and the effects of treatment. If the validity of the underlying assumptions is uncertain, clinicians should exercise caution when interpreting these studies; and further evaluation of the test remains ethical and essential.

## Competing interests

The authors declare that they have no competing interests.

## Authors' contributions

LPS developed the research proposal, selected the research methods, interpreted the findings of the literature review, and drafted the manuscript. SJL conceived the research idea, contributed to the selection of research methods and interpretation of the findings, and participated in the drafting of the manuscript. RJS helped develop the research question, participated in the interpretation of findings, and contributed to the drafting of the manuscript. SD participated in the selection of research methods, performed the literature review, and contributed to the interpretation of findings and drafting of the manuscript. NH and RYMC contributed to the interpretation of clinical examples and drafting of the manuscript. LI provided guidance for the interpretation of findings, and contributed to the drafting of the manuscript. All authors read and approved the final manuscript.

## Pre-publication history

The pre-publication history for this paper can be accessed here:

http://www.biomedcentral.com/1471-2288/12/12/prepub
